# High Mobility Group Box 1 and TLR4 Signaling Pathway in Gnotobiotic Piglets Colonized/Infected with *L. amylovorus*, *L. mucosae*, *E. coli* Nissle 1917 and *S*. Typhimurium

**DOI:** 10.3390/ijms20246294

**Published:** 2019-12-13

**Authors:** Igor Splichal, Sharon M. Donovan, Vera Jenistova, Iva Splichalova, Hana Salmonova, Eva Vlkova, Vera Neuzil Bunesova, Marek Sinkora, Jiri Killer, Eva Skrivanova, Alla Splichalova

**Affiliations:** 1 Laboratory of Gnotobiology, Institute of Microbiology, Czech Academy of Sciences, 549 22 Novy Hradek, Czech Republic; splichal@gnotobio.cz (I.S.); jenistova@gnotobio.cz (V.J.); marek@biomed.cas.cz (M.S.); 2 Department of Food Science and Human Nutrition, University of Illinois, Urbana, IL 61801, USA; sdonovan@illinois.edu; 3 Laboratory of Immunobiology, Institute of Molecular Genetics, Czech Academy of Sciences, 142 20 Prague, Czech Republic; iva.splichalova@img.cas.cz; 4 Department of Microbiology, Nutrition and Dietetics, Faculty of Agrobiology, Food and Natural Resources, Czech University of Life Sciences in Prague, 165 00 Prague, Czech Republic; salmonova@af.czu.cz (H.S.); vlkova@af.czu.cz (E.V.); bunesova@af.czu.cz (V.N.B.); killer@iapg.cas.cz (J.K.); skrivanovae@af.czu.cz (E.S.); 5 Laboratory of Anaerobic Microbiology, Institute of Animal Physiology and Genetics, Czech Academy of Sciences, 142 20 Prague, Czech Republic

**Keywords:** *Escherichia coli* Nissle 1917 (EcN), gnotobiotic piglet, high mobility group box 1 (HMGB1), intestine, *Lactobacillus amylovorus* (LA), *Lactobacillus mucosae* (LM), *Salmonella* Typhimurium (ST), Toll-like receptor 4 (TLR4)

## Abstract

High mobility group box 1 (HMGB1) is a DNA-binding nuclear protein that can be actively secreted by immune cells after different immune stimuli or passively released from cells undergoing necrosis. HMGB1 amplifies inflammation, and its hypersecretion contributes to multiple organ dysfunction syndrome and death. We tested possible immunomodulatory effect of commensal *Lactobacillus amylovorus* (LA), *Lactobacillus mucosae* (LM) or probiotic *Escherichia coli* Nissle 1917 (EcN) in infection of gnotobiotic piglets with *Salmonella* Typhimurium (ST). Transcription of HMGB1 and Toll-like receptors (TLR) 2, 4, and 9 and receptor for advanced glycation end products (RAGE), TLR4-related molecules (MD-2, CD14, and LBP), and adaptor proteins (MyD88 and TRIF) in the ileum and colon were measured by RT-qPCR. Expression of TLR4 and its related molecules were highly upregulated in the ST-infected intestine, which was suppressed by EcN, but not LA nor LM. In contrast, HMGB1 expression was unaffected by ST infection or commensal/probiotic administration. HMGB1 protein levels in the intestine measured by ELISA were increased in ST-infected piglets, but they were decreased by previous colonization with *E. coli* Nissle 1917 only. We conclude that the stability of HMGB1 mRNA expression in all piglet groups could show its importance for DNA transcription and physiological cell functions. The presence of HMGB1 protein in the intestinal lumen probably indicates cellular damage.

## 1. Introduction

High mobility group box 1 (HMGB1) is an intracellular nuclear DNA-binding protein that can be produced by innate immune cells or released from cells undergoing necrosis [1]. This evolutionarily conserved protein shows high interspecies amino acid homology [2] and participates in different processes, including transcription, replication, nucleosome formation, and tissue repair [3]. It is essential for life, as it was documented in mouse pups with deleted HMGB1 that were born alive, but died within 24 h [4]. HMGB1 belongs to damage-associated molecular patterns (DAMPs) called alarmins. The alarmins are endogenous intracellular factors that are normally hidden from immune recognition, but in some conditions, such as cellular stress or injury, they can be released to the cell vicinity and sensed [1,5,6]. Circulating HMGB1 arises from a combination of both active secretion and passive release from cells of different lineages [7]. It can either promote beneficial tissue repair and provoke deleterious uncontrolled inflammation [8].

Gram-positive and Gram-negative bacteria induce different inflammatory cytokine patterns [9] and their levels are higher in septic non-survivors compare to survivors [10]. HMGB1 shows cytokine activity [1]. It is released later in infections compared to inflammatory cytokines, as tumor necrosis factor (TNF)-α and interleukin (IL)-1β [11]. The exaggerated secretion/release of HMGB1 has a detrimental effect on surviving patients with sepsis [12]. The active secretion of HMGB1 undergoing to changes (acetylation, phosphorylation, and methylation) [13,14,15] and its passive release [16] can amplify innate immune response to multiple organ dysfunction syndrome and death [11,17]. Therefore, the increased levels of HMGB1 predict multiple organ dysfunction syndrome (MODS) with fatal consequences of infection [17]; thus, increased systemic HMGB1 is considered a biomarker of sepsis [11].

In contrast to DAMPs, pathogen-associated molecular patterns (PAMPs) are molecular structures typical for microorganisms [18]. Both PAMPs and DAMPs are recognized by pattern recognition receptors (PRRs) [19]. Toll-like receptors (TLRs) are one of the PRRs groups. TLR2, 4, and 9 recognize typical bacterial structures as well as HMGB1 [19,20,21]. A receptor for advanced glycation end (RAGE) is another HMGB1 recognizing receptor [5]. The shared recognition of PAMPs and DAMPs by the same receptors leads to similar activations and consequences in infections and sterile tissue traumas of various origins [22,23]. The need to re-evaluate old definition of sepsis [24] and update it [25] based on these novel molecular findings.

Closely related human and pig anatomy, genetics, physiology [26], and highly similar composition of microbiome [27] predetermine the pig as an animal model of human infectious [28] and gastroenterological diseases [29]. *Salmonella enterica* serovar Typhimurium (*S*. Typhimurium) commonly causes gastroenteritis (salmonellosis) in humans and pigs [30] but human typhoid fever-like illness in mice [31]. *S.* Typhimurium can also cause life-threatening invasive diseases in immunocompromised individuals [32]. The intracellular environment and frequent multidrug resistance protect *Salmonella enterica* against extracellular antibiotics and facilitates disease relapse [33,34,35]. Thus, it is necessary to look for alternative ways to combat infections with this foodborne pathogen [36,37]. One possibility is the modulation of the GIT microbiota by commensal and probiotic bacteria [38]. 

*Lactobacillus* spp. are Gram-positive facultative anaerobes that create an abundant bacterial group in human and pig microbiota in the distal small intestine and colon [39,40] . A strain-specific beneficial effect of lactobacilli is determined by high variability in composition of cell wall polysaccharides, peptidoglycan, and teichoic acids, membrane lipoproteins and lipoteichoic acids that can differentially induce the host immune response [41]. Moreover, all *Lactobacillus* spp. produce organic acids with antimicrobial properties and some species also produce other antimicrobial compounds, such as bacteriocins and H_2_O_2_ [42]. Despite the fact that *Lactobacillus* spp. are typically beneficial for the host, care should be taken with their application in immunocompromised hosts [43] and all new probiotic bacteria should be tested for their antimicrobial susceptibility [44]. Some lactobacilli strains, such as *L. rhamnosus* GG, *L. casei* Shirota, and *L. acidophilus* LB, are widely used probiotics [45], and commensal lactobacilli strains have been used to combat enteric pathogens [46,47]. Another abundant bacterial group in the intestinal tract are Gram-negative *Escherichia coli* that includes both pathogenic [48] and probiotic [49] strains. A probiotic *E. coli* Nissle 1917 (EcN) is anti-diarrheic in humans [50] and pigs [51]. This effect of EcN is mediated mainly by the production of colicins, microcins, and siderofores, and other systems for iron uptake [52]. 

Gnotobiotic animals have, in contrast to conventional animals, simple and defined microbiota [53]. Their lowered colonization resistance [54] suggests these animal models for the study of microbe versus host interactions with less virulent microbes that would be normally suppressed in the presence of a balanced microbiota [55]. Moreover, colostrum-free piglets are immunocompromised by deprivation of maternal immunoglobulins and cells [56]. Therefore, germ-free surgery-derived piglets that are reared in microbiologically controlled conditions can be used as a model of vulnerable immunocompromised infants [57]. *Salmonella* Typhimurium strain LT2 is a well-characterized laboratory strain [58]. It was safe for one-week-old conventional piglets after its oral application [59], but it caused gastroenteritis and sepsis in immunocompromised germ-free piglets of the same age [60]. 

Our work aimed to describe intestinal HMGB1 release after infection of gnotobiotic piglets with Gram-negative enteric pathogen *Salmonella* Typhimurium, signaling via TLR2, TLR4, TLR9, and RAGE, and the possible influence of previous colonization of the piglets with commensal Gram-positive lactobacilli *Lactobacillus amylovorus* and *Lactobacillus mucosae* or probiotic Gram-negative *E. coli* Nissle 1917. Knowledge of innate immune regulations and possibilities to modify TLRs signaling pathways can be helpful for the development of advanced therapeutic procedures to ameliorate health problems in the *S*. Typhimurium infections, especially in life-threatened immunocompromised individuals. 

## 2. Results

### 2.1. mRNA Relative Expressions of TLR4 and Its Related Molecules, TLR2, TLR9, and RAGE, in the Ileum

Eight groups of gnotobiotic piglets were used in the experiments (Supplementary Figure S1)—(i) germ-free (GF); (ii) GF piglets infected with *S.* Typhimurium (ST); (iii) GF piglets colonized with *L. amylovorus* (LA); (iv) LA piglets infected with *S.* Typhimurium (LA+ST); (v) GF piglets colonized with *L. mucosae* (LM); (vi) LM piglets infected with *S.* Typhimurium (LM+ST); (vii) GF piglets colonized with *E. coli* Nissle 1917 (EcN), and (viii) EcN piglets infected with *S.* Typhimurium (EcN+ST).

**Figure 1:**
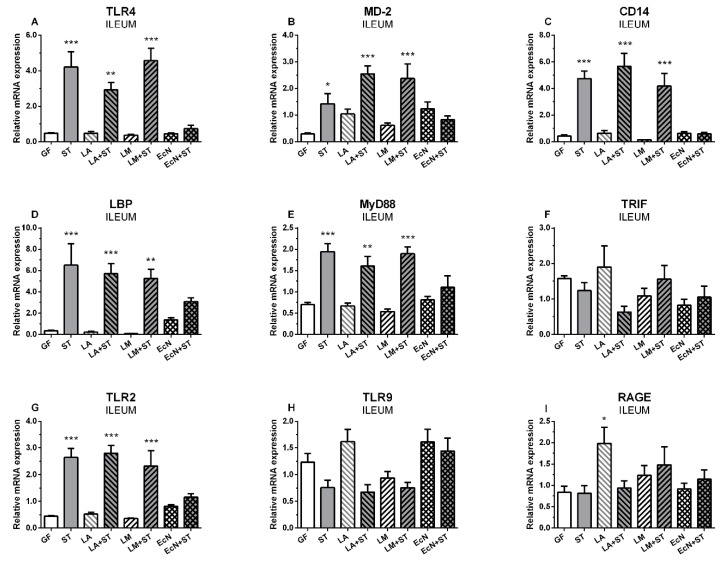
mRNA relative expression (fold change) of TLR4 and its related molecules, TLR2, TLR9, and RAGE in the ileum. TLR4 (**A**), MD-2 (**B**), CD-14 (**C**), LBP (**D**), MyD88 (**E**), TRIF (**F**), TLR2 (**G**), TLR9 (**H**), and RAGE (**I**) are presented as mean + SEM. Differences between the control GF group and other groups were evaluated by one-way analysis of variance (ANOVA) with Dunnett’s multiple comparison post-hoc test and depicted * *p* ˂ 0.05, ** *p* ˂ 0.01, *** *p* ˂ 0.001; *n* = 6 for each group.

Neither commensal *L. amylovorus* (LA) and *L. mucosae* (LM) nor probiotic *E. coli* Nissle 1917 (EcN) influenced TLR4 transcription in the ileum of the gnotobiotic piglets (Figure 1A). *S.* Typhimurium significantly upregulated mRNA expression of TLR4 in the ST, LA+ST, and LM+ST groups, but not in the EcN+ST piglets. LA, LM, and EcN slightly upregulated transcriptions of MD2 (Figure 1B) compare to the GF piglets. The upregulation was statistically significant in the ST, LA+ST, and LM+ST, but not in the EcN+ST group. Similarly to TLR4, a statistically significant upregulation of CD14 was induced by *S.* Typhimurium (Figure 1C), but previous colonization with *E. coli* Nissle 1917 prevented this upregulation. Comparable results were observed in lipopolysaccharide binding protein (LBP) (Figure 1D). Statistically significant upregulation of MyD88 expression was observed in the ST, LA+ST and LM+ST groups, but not the EcN+ST group (Figure 1E). In contrast, this was not observed in TRIF (Figure 1F), where no statistical significance between the GF and other groups was found. The TLR2 expression (Figure 1G) showed a similar pattern and statistical significance as to TLR4. In contrast to TLR2 and TLR4, no effect was observed in TLR9 expression (Figure 1H), and all groups were similar to the GF group. RAGE (Figure 1I) also did not show any obvious trend, and only the LM group only was statistically different from the control GF group.

### 2.2. mRNA Relative Expressions of TLR4 and Its Related Molecules, TLR2, TLR9, and RAGE, in the Colon

**Figure 2:**
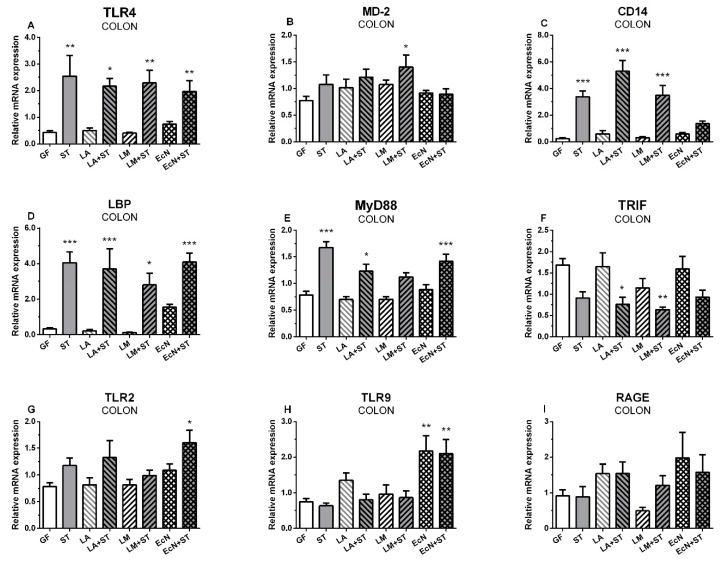
mRNA relative expression (fold change) of TLR4 and its related molecules, TLR2, TLR9, and RAGE in the colon. TLR4 (**A**), MD-2 (**B**), CD-14 (**C**), LBP (**D**), MyD88 (**E**), TRIF (**F**), TLR2 (**G**), TLR9 (**H**), and RAGE (**I**) are presented as mean + SEM. Differences between the control GF group and other groups were evaluated by one-way analysis of variance (ANOVA) with Dunnett’s multiple comparison post-hoc test and depicted * *p* ˂ 0.05, ** *p* ˂ 0.01, *** *p* ˂ 0.001; *n* = 6 for each group.

The relative mRNA expression of TLR4 in the colon (Figure 2A) was upregulated in all *S.* Typhimurium infected groups (ST, LA+ST, LM+ST, and EcN+ST) compared to GF group. MD2 showed statistically significant upregulation in LM+ST only (Figure 2B). TLR4 coreceptor CD14 (Figure 2C) showed significantly higher mRNA expression in all *Salmonella*-infected piglets ST, LA+ST, and LM+ST, except those formerly colonized with probiotic *E. coli* (EcN+ST). In contrast, LBP expression (Figure 2D) was upregulated in all *Salmonella*-infected groups without any exception. MyD88 (Figure 2E) and TRIF (Figure 2F) expression showed opposite trends and also showed different expression profiles than in the ileum. While MyD88 expression (Figure 2E) was significantly upregulated in the ST, LA+ST, and EcN+ST groups, TRIF expression was reduced (Figure 2F) in all *Salmonella*-infected groups, but was statistically significant downregulated in the groups previously colonized with lactobacilli (LA+ST and LM+ST) only. TLR2 did not show any obvious pattern (Figure 2G) except that expression was significantly greater in the EcN+ST group compared to the GF control. The TLR9 expression (Figure 2H) was statistically significantly upregulated in the groups colonized with EcN (EcN and EcN+ST) only. RAGE as TLR2 did not show any obvious trend (Figure 2I).

### 2.3. mRNA Relative Expressions of TLR4 and Its Related Molecules, TLR2, TLR9, and RAGE in Mesenteric Lymph Nodes

**Figure 3:**
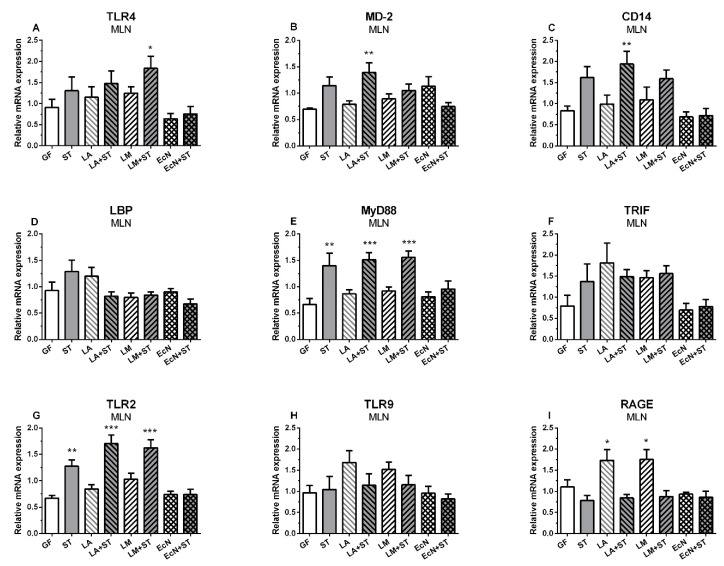
mRNA relative expression (fold change) of TLR4 and its related molecules, TLR2, TLR9, and RAGE in the MLN. TLR4 (**A**), MD-2 (**B**), CD-14 (**C**), LBP (**D**), MyD88 (**E**), TRIF (**F**), TLR2 (**G**), TLR9 (**H**), and RAGE (**I**) are presented as mean + SEM. Differences between the control GF group and other groups were evaluated by one-way analysis of variance (ANOVA) with Dunnett’s multiple comparison post-hoc test and depicted * *p* ˂ 0.05, ** *p* ˂ 0.01, *** *p* ˂ 0.001; *n* = 6 for each group.

The relative expression of TLR4 was upregulated in mesenteric lymph nodes (MLN) of the groups infected with *Salmonella* (ST, LA+ST, and LM+ST), but only in the group precolonized with *L. mucosae* was this upregulation statistically significant (Figure 3A). MD-2 was increased in the LM+ST group only (Figure 3B). The groups infected with *Salmonella,* including both groups preassociated with lactobacilli (ST, LA+ST, and LM+ST), showed increased expression of CD14 mRNA, but it was statistically significant in the LA+ST group only (Figure 3C). No statistically significant differences were found in LBP (Figure 3D). All *Salmonella*-infected groups (ST, LA+ST, and LM+ST) showed MyD88 mRNA statistically significant upregulation (Figure 3E). In contrast, no significant differences were found in another adaptor protein TRIF (Figure 3F). TLR2 mRNA was significantly upregulated in the *Salmonella*-infected groups (ST, LA+ST, and LM+ST) except the EcN+ST piglets (Figure 3G). TLR9 showed no significant changes in any group (Figure 3H). RAGE was significantly upregulated in both groups colonized with the commensal *L. amylovorus* or *L. mucosae* (Figure 3I). 

### 2.4. mRNA Relative Expressions of TLR4 and Its Related Molecules, TLR2, TLR9, and RAGE, in the Ileum, Colon, and MLN

**Figure 4:**
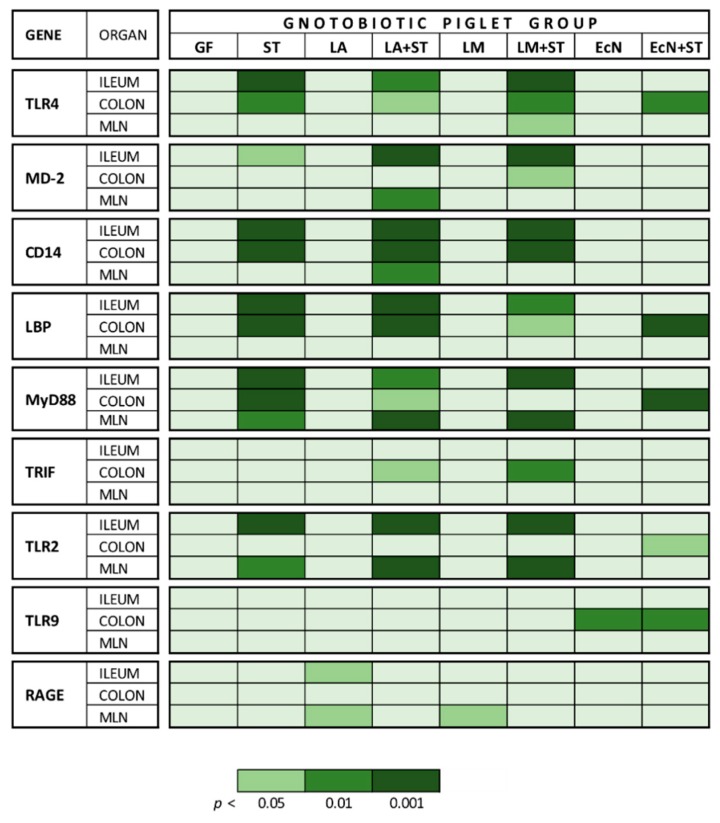
Summarized mRNA relative expression of TLR4 and its related molecules, TLR2, TLR9, and RAGE in the ileum, colon, and MLN. Differences between the control GF group and other groups were evaluated by one-way analysis of variance (ANOVA) with Dunnett’s multiple comparison post-hoc test and depicted * *p* ˂ 0.05, ** *p* ˂ 0.01, *** *p* ˂ 0.001; *n* = 6 for each group.

The mRNA relative expressions of TLR4 and its related molecules, TLR2, TLR9, and RAGE in the ileum, colon, and MLN were summarized in Figure 4.

### 2.5. mRNA Relative Expressions of HMGB1 in the Ileum, Colon, and MLN

**Figure 5:**
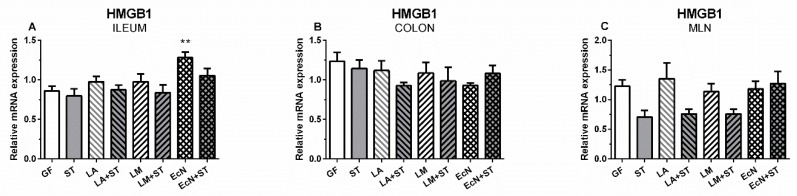
mRNA relative expression (fold change) of HMGB1 in the ileum, colon, and MLN of the gnotobiotic piglets. mRNA HMGB1 in the ileum (**A**), colon (**B**), and MLN (**C**) are presented as mean + SEM. Differences between the control GF group and other groups were evaluated by one-way analysis of variance (ANOVA) with Dunnett’s multiple comparison post-hoc test and depicted * *p* ˂ 0.05, ** *p* ˂ 0.01, *** *p* ˂ 0.001; *n* = 6 for each group.

In the ileum, the relative mRNA of HMGB1 differed (*p* ˂ 0.01) between the control GF and EcN groups only (Figure 5A). No significant differences were found in the colon (Figure 5B). mRNA HMGB1 expression in MLN (Figure 5C) was downregulated by *Salmonella* in the ST, LA+ST, and LM+ST groups, but did not reach the level of statistical difference compared to the GF control.

### 2.6. Cellular HMGB1 Protein in MLN

**Figure 6:**
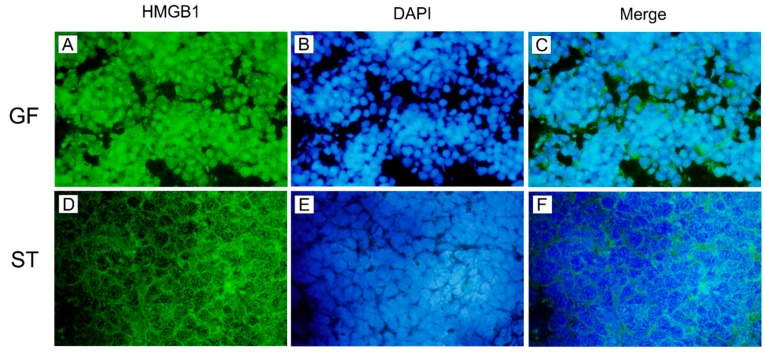
Immunofluorescence staining of HMGB1 in piglet MLN tissue. HMGB1 was stained green in the germ-free (GF; **A**) and *Salmonella* Typhimurium-infected piglets (ST; **D**) by Alexa 488. The nuclei were stained by blue in the GF (**B**) and ST piglets (**E**) by DAPI. HMGB1 was localized mainly in the nucleus in the GF piglets (**C**), but mainly in the cytoplasm in the ST group (**F**). Magnification is 1000×.

HMGB1 protein localization in the MLN tissue of the control GF piglets and *Salmonella*-infected piglets (ST) were studied by immunofluorescence (Figure 6). HMGB1 was localized mainly in nuclei of MLN cells in the GF group (Figure 6A–C). In the case of the ST group, HMGB1 translocated from the nucleus, and it was located mainly in the cytoplasm (Figure 6D–F).

### 2.7. Local and Systemic Levels of HMGB1

**Figure 7:**
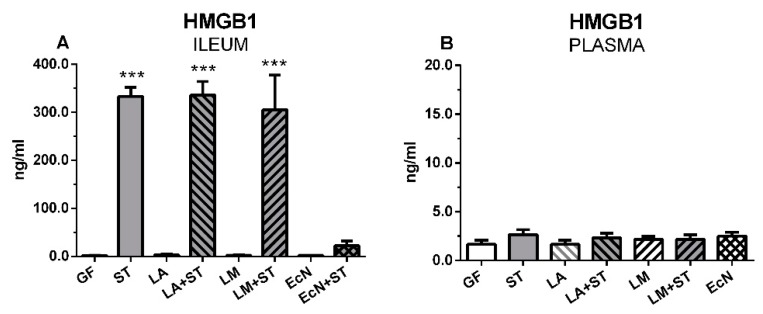
Local and systemic levels of HMGB1. The local HMGB1 levels in the ileum (**A**) and systemic HMGB1 levels in plasma (**B**) were measured in the gnotobiotic piglets by ELISA. Results are expressed as mean + SEM. Differences between the control GF group and other groups were evaluated by one-way analysis of variance (ANOVA) with Dunnett’s multiple comparison post-hoc test and depicted * *p* ˂ 0.05, ** *p* ˂ 0.01, *** *p* ˂ 0.001; *n* = 6 for each group.

Statistically significant increases of HMGB1 levels in the ileal lavages (Figure 7A) were found in the *Salmonella*-infected groups with ST, LA+ST, and LM+ST, with the exception of the piglets preliminary colonized with *E. coli* Nissle 1917 (EcN + ST). In plasma, much lower concentrations were detected compared to ileal contents and no statistically significant differences were found in any group (Figure 7B).

## 3. Discussion

LPS (endotoxin) is highly immunomodulatory compound that can induce endotoxin shock [61,62], which is associated with excessive secretion of inflammatory cytokines called “cytokine storm” and can provoke lethal multiple organ dysfunction syndrome (MODS) [17,63]. While IL-1 and TNF-α are early induced inflammatory cytokines, HMGB1 that was the object of our interest is a late mediator of inflammatory reaction [11]. TLR4 is the common point of LPS and HMGB1 signaling pathways, because TLR4 is the receptor for both molecules [5,21] and HMGB1 potentiates the inflammatory effects of LPS [64]. The modulation of the TLR4 pathway can mitigate the injury from Gram-negative bacteria-induced sepsis. Signalling through TLR4 can be modulated by treatment with low-molecular weight natural and synthetic compounds [65], neutralizing antibodies [66,67,68], decoy soluble receptors [69], natural products and their derivatives [70], and probiotics [66,71,72]. The modulation can be targeted directly to TLR4 or its related molecules as co-receptors or adaptor proteins [73]. Released LPS [74,75] binds to LPS-binding protein (LBP) in serum, and the newly created LPS-LBP complex binds to CD14, which is expressed mainly on the cell membrane of phagocyting cells, but it also on other cell types, including enterocytes. The secreted MD-2 is associated with TLR4 and is essential for the expression of TLR4 and responsiveness of TLR4 to LPS [20,73].

*Lactobacillus* spp. are abundant in pig distal small intestine and colon [39,76]. Previously, selected lactobacilli were shown to modulate the TLR4 signaling in intestinal cell lines [72,77], intestinal explants [72], and conventional pigs [78]. We attempted to modulate the TLR4 signaling pathway in the gnotobiotic piglets using two commensal lactobacilli species, *L. amylovorus* and *L. mucosae,* and the probiotic *E. coli* Nissle 1917. We anticipated that the actions of two *Lactobacillus* strains would differ. It was reported that *L. amylovorus* modulation of TLR4 signaling prevented damage of an epithelial cell line and intestinal explants infected with enterotoxigenic *E. coli* K88 [72,79], whereas *L. mucosae* express a typical strain-specific mucus-binding protein (Mub), which mediates its binding to mucus in vitro [80]. *E. coli* Nissle 1917 has a semi-rough LPS (incompletely synthesized LPS chain) that determines its serum sensitivity and prevents the immunocompetent host from developing bacteremia from *E. coli* Nissle 1917 [81].

The main site of cross-talk between *Salmonella* Typhimurium and the host is in the distal ileum [29]. It is also the gastrointestinal segment with a plentiful population of aerotolerant bacteria in humans and animals, including lactobacilli and *E. coli* [76,82]. *S.* Typhimurium infection in gnotobiotic piglets up-regulated ileal mRNA expression of both members of TLR4/MD-2 complex and its co-receptor CD14 and LBP, with the exception the piglets previously associated with *E. coli* Nissle 1917 . This increase in TLR4 expression coincided with an upregulation of chemokine IL-8, pro-inflammatory cytokine TNF-α, and regulatory cytokine IL-10 in *S*. Typhimurium-infected piglets, but not in the piglets associated with *L, amylovorus*, *L. mucosae* or *E. coli* Nissle 1917, as we described elsewhere [47]. Thus, previous colonization with *E. coli* Nissle 1917 suppressed the increased cytokine expression induced by *S*. Typhimurium infection. 

It was shown that the avirulent rough LPS *Salmonella enterica* serovar Infantis and serovar Typhimurium, with an incompletely synthesized LPS chain, both protected gnotobiotic piglets against subsequent infection with virulent *S*. Typhimurium strains F98 [83] and LT2 [84], respectively. The protective effect may be due to local production of IL-8 and subsequent neutrophil recruitment [83,84], actions on the TLR4 signaling pathway changes had not been previously reported. We speculate that rough LPS of both *S*. *enterica* serovars [83,84], as well as semi-rough LPS of *E. coli* Nissle 1917 [47], disrupted the TLR4/MD-2 signaling pathway and inhibited its ability to be triggered by the smooth LPS of the virulent *S*. Typhimurium. Therefore, they prevented the excessive detrimental production of the inflammatory cytokines [47]. All the bacteria mentioned above, which have incompletely synthesized LPS, induced physiological levels of IL-8 [47,83,84], which is necessary for the attraction and activation of neutrophils to the site of infection. 

TLR4 is only one of the Toll-like receptors that use both MyD88 and TRIF adaptor molecule pathways [20,85]. MyD88 and TRIF are responsible for the activation of distinct signaling pathways, leading to the production of pro-inflammatory cytokines and type I interferons, respectively [5,73]. The LPS structure completeness influences the activation of the TLR4 signaling via MyD88 and TRIF pathways [85]. While MyD88 mRNA expression patterns followed those of TLR4, MD-2, and CD14 in the ileum, the expression of TRIF did not show any obvious relation to the infection with *S.* Typhimurium or association to other bacteria used before the challenge with *S.* Typhimurium. We concluded that the signaling in the *Salmonella* infection was via the MyD88 regulatory pathway. 

The CD14 is well known about as co-receptor of TLR4, but much less is known its participation as co-receptor of TLR2 [86] and TLR9 [87]. The disruption of TLR signaling pathway induced by live virulent *E. coli* and *E. coli*-derived LPS in pigs by CD14 neutralization antibodies resulted in decreased levels of pro-inflammatory cytokines IL-1β, IL-6, IL-8, and TNF-α, and lower granulocyte activation in a pig model of sepsis [88]. TLR2 mRNA showed the same trend as the TLR4/MD-2 complex members in the ileum. In contrast, TLR9 mRNA expression was downregulated by *S*. Typhimurium in the ileum, but the downregulation was not statistically significant. It is possible that TLR2 and TLR9 play opposite roles during *S*. Typhimurium infection in the gnotobiotic piglets, as was found in mice [89]. Consistent with the TLR2 and TLR4, previous association with the probiotic *E*. *coli* Nissle 1917 inhibited the up-regulation of TLR9 mRNA expression after *S*. Typhimurium infection. Similarly, *E. coli* Nissle 1917 ameliorated dextran sulfate-induced colitis and decreased the pro-inflammatory cytokine response in wild-type mice, but not in TLR2 or TLR4 knockout mice [90], which supports *E. coli* Nissle 1917 immunomodulation of host TLR2 and TLR4 signaling. Concordantly with our findings of induced inflammatory cytokines reported elsewhere [47], 4-week-old weaned conventional piglets orally-infected with *S*. Typhimurium showed upregulation of TLR2 and TLR4 in the ileum that resulted in the expression of pro-inflammatory cytokine IL-1β, IL-6, and TNF-α mRNA expressions 2 days post-infection [91]. 

The colon has a different histological structure and physiological role than the ileum [92]. The microbiota in the colon are composed of highly abundant anaerobes, and a lower proportion of lactobacilli and other aerotolerant bacteria compared to the ileum [76,93]. It was reported that different parts of the conventional piglet intestine infected with *S*. Typhimurium differed in their mRNA expression of inflammatory response-related genes, including TLRs [94]. In the colon, we found similar trends in TLR4, CD14, LBP, MyD88 as in the ileum, but not in the case of TLR2, which did not show any obvious trend. Both *E*. *coli* Nissle 1917-associated groups showed significantly increased TLR9 mRNA expression, which has not been previously reported.

The MLN are the main site of antigen presentation to combat bacteria that are translocated via M-cells, enterocytes, and dendritic cells or have penetrated autonomously through the disrupted intestinal barrier [95,96]. The translocated bacteria are destroyed immediately in the MLN, or their systemic spread is delayed. The processing of bacterial antigens or delays in systemic spread allows the establishment of a more efficient adaptive immune response [97,98]. TLR2 and TLR9 mRNA and proteins were expressed in the ileum, and MLN of newborn healthy piglets, with much higher expression in the MLN [99]. These findings support the ability of the piglets to respond to TLR ligands from birth. 

In our experiments, gene expression of the receptors was lower in the MLN than either ileum or colon. In the MLN, expression of CD14, TLR2, and MyD88 all increased in response to *Salmonella*-infection, except in piglets previously innoculated with the probiotic *E. coli*. MD-2 enables TLR-2 to recognize LPS and enhances TLR2-mediated responses to both Gram-positive and Gram-negative bacterial, including peptidoglycan, lipoteichoic acid components, and LPS [100]. It seems that Gram-negative *Salmonella* was probably discriminated in the cooperation of MD-2, CD14, TLR4 but also TLR2. The TLR2 signaling confirmed increased MyD88 mRNA expression that is the adaptor molecule participating in the TLR2 signaling pathway [73].

In contrast to our results, four-week-old conventional piglets orally infected with 10^8^ CFU *S*. Typhimurium serovar DT104 did not show any differences in CD14, TLR2, and MyD88 mRNA expression in the MLN two days post-infection [101]. RAGE mRNA was significantly increased in both groups of the piglets mono-associated with both commensal lactobacilli. However, RAGE is a multiligand receptor, including HMGB1, and exhibits various functions in physiological and pathological processes [5,102]. The reason of this upregulation is unknown, especially in the case of *L. amylovorus,* which was not found in the MLN, in contrast to *L. mucosae* that was found in a low number of CFUs in part of the piglets [47]. Even in the absence of live lactobacilli, there is possible stimulation by their processed PAMPs or DAMPs, such as as HMGB1 [5]. The elucidation of the commensal lactobacilli-induced upregulation in the MLN will need additional experiments.

HMGB1 is the endogenous ligand of TLR2, TLR4, and TLR9 [5]. It is an important inflammatory marker in the intestine in different pathologies. It was secreted into intestinal lumen either in enteric infections [103] or in inflammatory bowel diseases [104]. Necrotizing enterocolitis (NEC) that affects the immature intestine shows its devastating consequences partially due to the high expression of TLR4, which results in the induction of inflammatory cytokines and impairment of the epithelial barrier [105]. This illness shares some common characteristics with salmonellosis: it affects the ileum and colon, and neutrophils are recruited into the affected tissues [106,107]. The severity of the NEC in the rat model positively correlated with TLR4 and HMGB1 expression [108]. High levels of HMGB1 were also found in the intestine and plasma of *E. coli* O55-infected gnotobiotic piglets, but not in infected piglets that thrived [109]. Gnotobiotic piglets with highly immature intestine also showed recruitment of inflammatory cells in the *S*. Typhimurium-infected ileum [47]. Lastly, intestinal TLR4 mRNA upregulation and HMGB1 protein levels were increased in both TLR4 and HMGB1 proteins in the pig ileal tissue 120 hrs after the infection with *Clostridium perfringens* type C [110]. Therefore, HMGB1 can serve as the marker of the damaged intestine.

Posttranslational modifications of HMGB1 determine its localization, migration, and biological activity. HMGB1 protein stays in the nucleus, but its hyperacetylation [13], hyperphosporylation [15], or mono-methylation [14] move HMGB1 from a nuclear localization to the cytoplasm [111]. After the exocytosis, the secreted HMGB1 may create complexes with LPS (similarly as LBP), transfer it to CD14, and initiate TLR4/MD-2-mediated inflammatory response and alongside increases HMGB1 magnitude [112]. We presented the micrographs of the mesenteric lymph nodes of the germ-free and *S*. Typhimurium-infected piglets only to simplify imagination about the HMGB1 localization and migration changes in the *S*. Typhimurium infection. HMGB1 in the mesenteric lymph nodes of the GF piglets was colocalized mainly with the nucleus, but it was spread into the cytoplasm in the *Salmonella*-infected ST group. 

HMGB1 translocated from the nucleus to the cytoplasm and was secreted from cells; nevertheless, the expression of HMGB1 mRNA was not upregulated 16 hrs after the stimulation [63]. The higher intestinal HMGB1 levels in the *S.* Typhimurium-infected groups suggest that secretion was in response to the infection. Lower intestinal HMGB1 levels in the intestine of infected piglets who has been inoculated with *E. coli* Nissle 1917 (EcN+ST) confirmed the anti-*Salmonella* properties of this probiotic bacteria [52]. The HMGB1 mRNA expression showed no significant differences among the piglet groups in our experiments. It indicates that increased intestinal HMGB1 levels in the *Salmonella*-infected piglets were not from de novo synthesized HMGB1.

In conclusion, HMGB1 mRNA transcription was not activated by the infection of the gnotobiotic piglets with *S*. Typhimurium within 24 hrs. This stability of the HMGB1 mRNA expression in all groups underlines the importance of HMGB1 for DNA transcription and other physiological cell functions and indicates that increased intestinal HMGB1 levels likely originated from formerly synthetized and actively secreted HMGB1 or passively released HMGB1 from damaged cells. However, the presence of HMGB1 protein in the intestinal lumen probably more attests spontaneous release after cellular damage than induced secretion as it was possible to suppress with the prior association of the gnotobiotic piglets with probiotic *E. coli* 1917. Future bacteria-driven immunomodulatory studies are needed to modify the signaling pathways and prevent or dampen the sepsis. 

## 4. Materials and Methods

### 4.1. Ethics Statement

All animal experiments were approved by the Animal Care and Use Committee of the Czech Academy of Sciences, protocol No. 117/2012, on 24 April 2012. 

### 4.2. Bacterial Suspensions 

All bacterial strains were from a collection of microorganisms at the Laboratory of Gnotobiology (Novy Hradek, Czech Republic). A probiotic *E. coli* Nissle 1917 (EcN) was originally donated to the collection by U. Sonnenborn (Ardeypharm, Herdecke, Germany) and *Salmonella enterica* serovar Typhimurium strain LT2 (ST) by O. Lüderitz (Max Planck Institute of Immunobiology, Freiburg, Germany). Commensal pig lactobacilli *L. amylovorus* strain P1 (LA) and *L. mucosae* strain P5 (LM) were isolated and characterized as we described elsewhere [47]. Fresh bacterial cultures were prepared by 16 hrs cultivation at 37 °C. EcN and ST grew on meat-peptone agar slopes (Oxoid, Basingstoke, UK) and lactobacilli in 10 mL MRS broth (Oxoid). Harvested cells were resuspended to an approximate 5 × 10^8^ CFU/m; in PBS.

Bacterial colonization of the gnotobiotic piglet intestine and translocation into liver, spleen, lungs, and blood we described elsewhere [47].

### 4.3. Gnotobiotic Piglets

Gnotobiotic piglets were obtained and bred in gnotobiotic isolators as described elsewhere [57]. Fifty-five piglets were divided to eight groups (Supplementary Figure S1): (i) GF (germ-free, *n* = 6); (ii) ST (one-week-old GF piglets orally infected with 1 × 10^6^ CFU of *S*. Typhimurium for 24 h, *n* = 7); (iii) LA (GF orally colonized with 1 × 10^8^ CFU of *L. amylovorus* 4 h after hysterectomy, *n* = 7); (iv) LA+ST (one-week-old LA-colonized piglets orally infected with 1 × 10^6^ CFU of *S.* Typhimurium for 24 h, *n* = 7); (v) LM (GF orally colonized with 1 × 10^8^ CFU of *L. mucosae* 4 h after hysterectomy, *n* = 7); (vi) LM+ST (one-week-old LM-colonized piglets orally infected with 1 × 10^6^ CFU of *S.* Typhimurium for 24 h, *n* = 7); (vii) EcN (GF orally colonized with 1 × 10^8^ CFU of *E. coli* Nissle 1917 4 h after hysterectomy, *n* = 7), and (viii) EcN+ST (one-week-old EcN-colonized piglets orally infected with 1 × 10^6^ CFU of *S.* Typhimurium for 24 h, *n* = 7). Each piglet group was composed of three independent hysterectomies and bred separately from other groups in a fiberglass gnotobiotic isolators. The bacteria were supplied in 5 mL of a milk diet, and the control GF piglets received 5 mL of milk without any bacteria. At the end of the experiment, the piglets were humanely euthanized by exsanguination via cardiac puncture under isoflurane anesthesia.

### 4.4. Total RNA Isolation, Reverse Transcription

Total RNA from the terminal ileum, transverse colon, and mesenteric lymph nodes was isolated and transcribed as described elsewhere [57]. Approximately 10 mg of the tissue stored in RNAlater (Qiagen, Hilden, Germany) at −20 °C were moved to RLT buffer of the RNeasy Plus Mini kit (Qiagen) with zirconia beads (BioSpec Products, Bartlesville, OK). The tissue was homogenized in TissueLyser LT beadbeater (Qiagen). The next steps followed the manufacturer’s instructions. Five hundred ng of the total RNA (A_260_/A_280_ ≥ 2.0 as measured in 10 mM Tris-HCl buffer pH 7.5) were reverse transcribed by QuantiTect Reverse Transcription kit (Qiagen) according to manufacturer’s instructions. 20 μL of the synthesized cDNA was 1/10 diluted by PCR quality water (Life Technologies, Carlsbad, CA), and these PCR templates were stored at −25°C until the following real-time PCR.

### 4.5. Real-Time PCR

2 μL of the PCR template was added to 18 μL of the FastStart Universal Probe Master (Roche Diagnostics) containing 100 nM LNA (lock nucleic acid) probe (Universal ProbeLibrary; Roche Diagnostics) and 500 nM each of the forward and reverse primers (Generi-Biotech, Hradec Kralove, Czech Republic)—Table 1.

Ten minutes’ initial heating at 95 °C was followed by 45 cycles at 95 °C for 15 s and 60 °C for 60 s were incubated and measured in duplicates on an iQ cycler with iQ5 Optical System Software 1.0 (Bio-Rad, Hercules, CA, USA). Cq for genes of the interest was normalized to Cq for β-actin and cyclophilin A reference genes and the relative mRNA fold change expressions of the genes of interest were calculated by 2^−Δ*C*T^ method [113] by GenEx 6.1 software (MultiD Analyses AB, Gothenburg, Sweden).

### 4.6. Immunofluorescent Detection of HMGB1 in Mesenteric Lymph Nodes

Mesenteric lymph nodes were embedded in Tissue-Tek (Sakura, Tokyo, Japan), snap-frozen in isopentane cooled in liquid nitrogen vapor, and stored at −70 °C. 5-μm acetone-fixed cryosections on SuperFrost/Plus slides (Thermo Fisher Scientific, Darmstadt, Germany) were kept at −40 °C until labeling. The sections were incubated with 10% normal rabbit serum (Life Technologies, Carlsbad, CA, USA) in a humid chamber for one h at RT. Labeling by anti-HMGB1 rabbit polyclonal antibodies (Novus Biologicals, Centennial, CO, USA) was performed overnight at 4 °C. The sections were incubated with secondary antibody, Alexa Fluor 488 goat anti-rabbit IgG (Life Technologies), for 2 h at RT. The sections were subsequently embedded in ProLong Gold Antifade Reagent (Life Technologies) and examined under an Olympus BX 40 microscope with a Olympus Camedia C-2000 digital camera (Olympus, Tokyo, Japan). Control sections without primary antibody were treated in the same way. The colocalization of HMGB1 and nuclei was analyzed by ImageJ software [114].

### 4.7. Local and Systemic HMGB1 Levels 

40 cm of the distal small intestine (labelled here as ileum) was filled with 2 mL of Dulbecco’s Phosphate Buffered Saline (DPBS), gently kneaded, and irrigated. The obtained intestinal lavages were spun at 2500× *g* for 30 min at 8 °C, and supernatants were filtered through a 0.2 μm nitrocellulose filter (Sartorius, Goettingen, Germany). A citrated blood sample was spun at 1200× *g* for 10 min at 8 °C. A protease inhibitor cocktail (Roche Diagnostics, Manheim, Germany) was added to the lavage supernatants and plasma. Both supernatants and plasma were aliquoted, immediately frozen, and stored at −45 °C. The HMGB1 levels were quantified in duplicates by ELISA kit (IBL International, Hamburg, Germany) according to manufacturer’s instructions. Optical densities were measured at 450 nm and 620 nm with an Infinite M200 Microplate reader and the results were evaluated with Magellan 6.3 software (Tecan, Grödig, Austria).

### 4.8. Statistical Analysis

Differences of the groups to the control GF group in parameters with normal distribution were evaluated with one-way ANOVA with Dunnet’s multiple comparisons post-hoc test. The statistical evaluations were performed at * *p* ˂ 0.05, ** *p* ˂ 0.01, and *** *p* ˂ 0.001 by GraphPad 6 software (GraphPad Software, La Jolla, CA, USA) and statistical significances of differences depicted in figures by asterisks.

## Figures and Tables

**Table 1:** LNA probe-based Real-Time PCR systems.

Gene	5′-Forward primer-3′	5′-Reverse primer-3′	#LNA Probe
BACT ^1^	TCCCTGGAGAAGAGCTACGA	AAGAGCGCCTCTGGACAC	9
CYPA ^2^	CCTGAAGCATACGGGTCCT	AAAGACCACATGTTTGCCATC	48
HMGB1	AGGAGAGCATCCTGGCCTA	ATCTGCAGCGGTGTTATTCC	9
TLR4 ^3^	CCATGGCCTTTCTCTCCTG	TCAGCTCCATGCATTGGTAA	33
MD-2 ^4^	GCTCTGAAGGGAGAGACTGTG	TTGTCCCGGAGAAAATCGTA	12
CD14 ^5^	TCTCACCACCCTGGACCTAT	AACTTGCGCGGACAGAGA	23
LBP ^6^	ACTAGACGGCTCCTTTGACG	GCCCAGGAGAAGATTGACTG	9
TLR2 ^3^	CTGCTCCTGTGACTTCCTGTC	AGGTAGTTCTCCGGCCAGTC	40
TLR9 ^3^	CAATGACATCCATAGCCGAGT	CGTTGCCGCTAAAGTCCA	3
MyD88 ^7^	GCAGCTGGAACAGACCAACT	GTGCCAGGCAGGACATCT	41
TRIF ^8^	ATCTCCCTGGAGGCACTGA	GCTGTCTACACCAGCCCACT	9

^1^ β-actin, ^2^ cyclophylin A, ^3^ Toll-like receptor, ^4^ myeloid differentiation protein 2, ^5^ cluster of differentiation 14, ^6^ lipopolysaccharide binding protein, ^7^ myeloid differentiation factor 88, ^8^ TIR-domain-containing adapter-inducing interferon-β.

## Supplementary Materials

Supplementary materials can be found at https://www.mdpi.com/1422-0067/20/24/6294/s1.

## Abbreviations

2^−Δ*C*T^	Comparative C_T_ method
BACT	β-actin
C_q_	Cycle of quantification
C_T_	Treshold cycle
CYPA	Cyclophylin A
DAMPs	Damage-Associated Molecular Patterns
DAPI	4′,6-diamidino-2-phenylindole
EcN	*Escherichia coli* Nissle 1917
ELISA	Enzyme Linked Immuno Sorbent Assay
GF	Germ-free
GIT	Gastrointestinal tract
HMGB1	High mobility group box 1
IFN	Interferon
IL	Interleukin
LA	*Lactobacillus amylovorus*
LBP	Lipopolysaccharide binding protein
LM	*Lactobacillus mucosae*
LNA	Locked nucleic acid
LT2	*Salmonella* Typhimurium strain LT2
MD-2	Myeloid differentiation factor 2
MRS	De Man, Rogosa, and Sharpe
MyD88	Myeloid differentiation primary response 88
NEC	Necrotizing enterocolitis
NF-κB	Nuclear factor kappa B
NTS	Non-typhoidal Salmonellae
PAMPs	Pathogen-Associated Molecular Patterns
PRRs	Pathogen Recognition Receptors
RAGE	Receptor for Advanced Glycation End
RT-qPCR	Real-Time quantitative Polymerase Chain Reaction
ST	Salmonella Typhimurium
TLR	Toll-like Receptor
TNF	Tumor Necrosis Factor
TRIF	TIR domain-containing adaptor inducing IFN-β
