# Supplementary figures and images for: High Mobility Group Box 1 and TLR4 Signaling Pathway in Gnotobiotic Piglets Colonized/Infected with L. amylovorus, L. mucosae, E. coli Nissle 1917 and S. Typhimurium
Source: Int J Mol Sci. 2019 Dec 13;20(24):6294. doi: 10.3390/ijms20246294 (PMC6940798; doi:10.3390/ijms20246294)

## SCHEMA OF EXPERIMENT WITH GNOTOBIOTIC PIGLETS

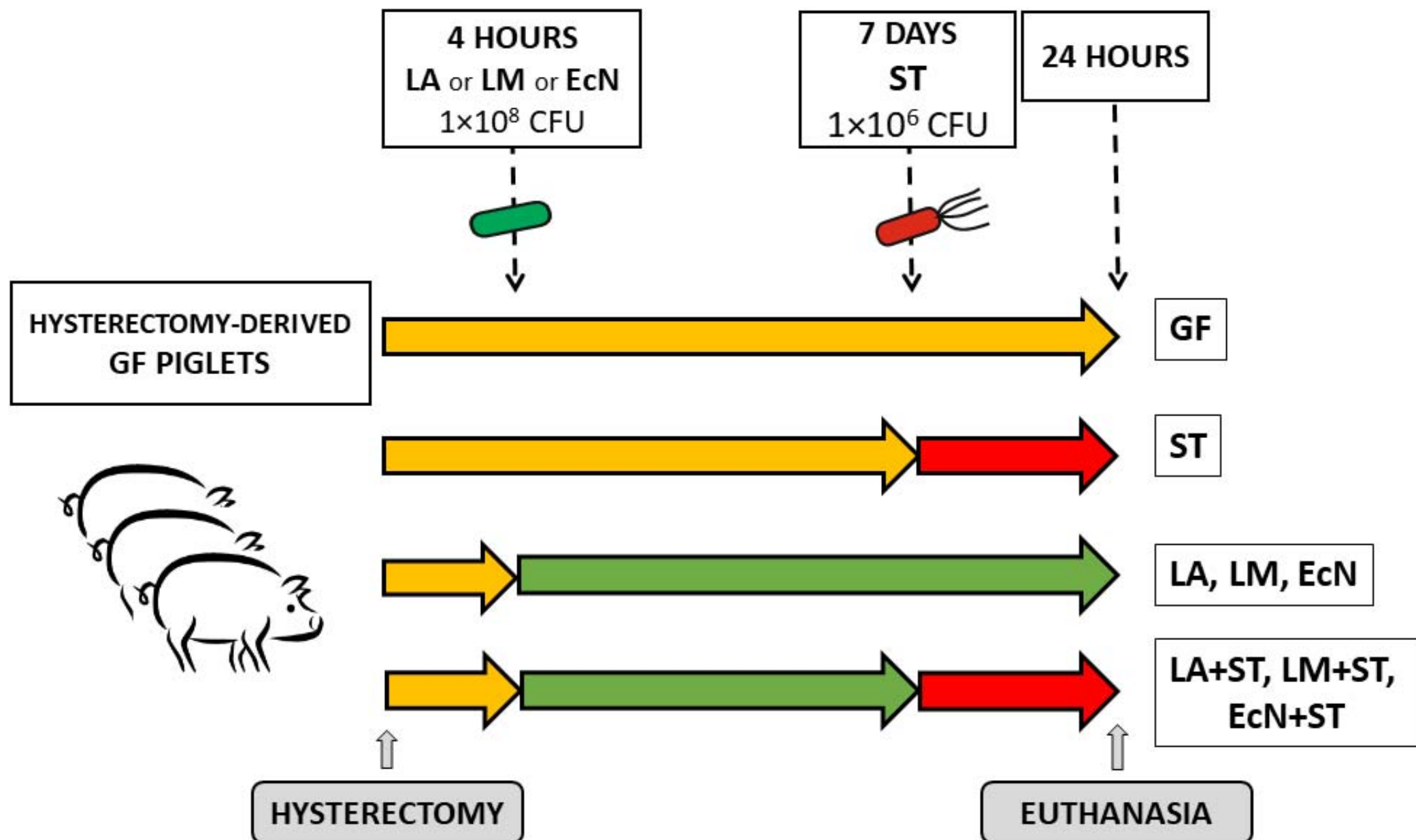

Supplement: Supplementary file 1 [file ijms-20-06294-s001.pdf]
